# Innovative gene targeted treatments for osteosarcoma: a mini review of current clinical evidence and future prospects

**DOI:** 10.3389/fmed.2025.1699287

**Published:** 2025-11-19

**Authors:** Dong Hu, Xingxing Yu, Junkui Xu, Bingbing Li, Xuehai Ou, Shaoyan Shi

**Affiliations:** 1 Honghui Hospital, Xi'an Jiaotong University, Xi'an, China; 2 Department of Laboratory Medicine, Xi'an Medical College, Xi'an, China

**Keywords:** osteosarcoma, tumor microenvironment, targeted therapy, mTOR pathway, tyrosine kinase inhibitors, precision oncology

## Abstract

Osteosarcoma is the most common primary malignant bone tumor in adolescents and young adults, marked by genomic instability and a high rate of lung metastasis. While surgery and intensive chemotherapy have improved survival for localized disease, outcomes for recurrent or metastatic cases remain poor, with limited progress in recent decades. In response, targeted therapies have emerged, focusing on key oncogenic pathways and tumor microenvironmental factors. Recent clinical studies have explored tyrosine kinase inhibitors (e.g., sorafenib, regorafenib), PI3K/Akt/mTOR inhibitors, angiogenesis modulators (e.g., apatinib), and immune checkpoint inhibitors. Although some agents achieve transient disease stabilization or partial responses, their overall efficacy is constrained by tumor heterogeneity, rapid resistance, and the lack of predictive biomarkers. Notably, combination regimens—such as VEGF and mTOR inhibition or TKI with immunotherapy—have shown promise in preclinical and early clinical trials. Future directions emphasize precision medicine approaches, including liquid biopsies and molecular profiling to guide therapy selection. Nanotechnology-based delivery systems are also under development to enhance tumor targeting and reduce systemic toxicity. However, the rarity of osteosarcoma, trial design limitations, and treatment-related toxicities remain critical barriers. This review synthesizes current evidence and underscores the need for biomarker-driven, multimodal strategies to overcome resistance and improve long-term outcomes in osteosarcoma management.

## 1 Introduction

Osteosarcoma constitutes the most prevalent form of malignant bone tumor and predominantly affects children, adolescents, and young adults. This neoplasm accounts for approximately 20% of all primary bone malignancies (1, 2). It is characterized by aggressive growth, significant genomic instability, and a high propensity for pulmonary metastasis, which remains the leading cause of mortality (3, 4). Clinically, osteosarcoma exhibits considerable heterogeneity at both genetic and histopathological levels, complicating diagnosis, risk stratification, and response prediction (5). Emerging evidence associates this heterogeneity with the tumor microenvironment (TME), where variable immune infiltration and stromal interactions are central drivers of resistance (3, 5–7). The standard treatment for osteosarcoma typically involves surgical resection in conjunction with multi-agent chemotherapy regimens, including methotrexate, doxorubicin, cisplatin, and ifosfamide, which have been employed since the 1980s. These interventions have increased survival rates for patients with localized disease to 60%–70%. However, individuals with recurrent, chemoresistant, or metastatic osteosarcoma continue to face poor long-term survival rates, which remain below 20% (8). Despite extensive research endeavors, overall survival rates have not markedly improved, highlighting the pressing need for innovative therapeutic strategies beyond traditional chemotherapy (9). Recent advancements in genetic and molecular research have elucidated critical pathways involved in osteosarcoma pathogenesis, such as the PI3K/Akt/mTOR axis, VEGF-mediated angiogenesis, and PD-1/PD-L1 signaling. Furthermore, elements of the TME, including immune suppression and stromal support, significantly affect therapeutic response (10). These insights have propelled the development of targeted agents aimed at inhibiting tumor proliferation, disrupting angiogenesis, or enhancing antitumor immunity. Various classes of targeted therapies are currently under investigation for osteosarcoma. Tyrosine kinase inhibitors disrupt signaling pathways that promote tumor growth and angiogenesis. mTOR inhibitors impede intracellular growth and survival mechanisms. Antiangiogenic agents inhibit the tumor vasculature, while immune checkpoint inhibitors aim to enhance antitumor immune responses (11). Clinical studies have demonstrated that these agents can occasionally induce disease stabilization or partial responses; however, their efficacy is generally limited and transient, primarily due to the emergence of resistance and the absence of reliable predictive biomarkers (12). Ongoing research is focused on elucidating the impact of intratumoral and microenvironmental heterogeneity on therapeutic outcomes. Contemporary strategies include combination regimens, biomarker-driven patient selection, and the development of advanced drug delivery platforms to achieve more durable clinical benefits (13, 14). This review synthesizes recent clinical evidence on targeted therapies for osteosarcoma. It encompasses the main results from clinical trials, elucidates the rationale behind different treatment strategies, and discusses current challenges, particularly how genetic and environmental factors in tumors affect treatment response (15).

## 2 Current targeted therapeutic approaches in osteosarcoma

Over the past decade, a variety of targeted therapeutic strategies have been investigated in osteosarcoma, aiming to disrupt tumor signaling, angiogenesis, and immune evasion. The most extensively studied pharmacological agents include tyrosine kinase inhibitors (TKIs), inhibitors of the PI3K/Akt/mTOR pathway, and angiogenesis modulators.

### 2.1 Tyrosine kinase inhibitors (TKIs)

TKIs inhibit multiple receptor tyrosine kinases involved in the growth, survival, and angiogenesis of osteosarcoma. Sorafenib, which targets VEGFR, PDGFR, and RAF, has demonstrated modest antitumor activity in heavily pretreated patients, primarily stabilizing the disease in a subset of individuals (16). When administered in combination with everolimus, it enhanced progression-free survival compared to sorafenib alone, indicating the potential of dual pathway inhibition (17). Regorafenib has demonstrated more consistent outcomes. In the SARC024 phase II trial, it significantly prolonged progression-free survival compared to placebo in patients with metastatic osteosarcoma (18), thereby establishing regorafenib as one of the most clinically validated tyrosine kinase inhibitors (TKIs). Cabozantinib, which targets MET and VEGFR2, has also shown efficacy in patients with refractory disease, including those with pulmonary metastases (19). Other TKIs, such as pazopanib and cediranib, have been investigated, resulting in partial responses and disease stabilization; however, durable results remain limited (20). Collectively, these trials suggest that TKIs can provide temporary disease control but are inhibited by tumor heterogeneity and the development of resistance (21).

### 2.2 PI3K/Akt/mTOR pathway inhibitors

The PI3K/Akt/mTOR signaling pathway is often dysregulated in osteosarcoma, facilitating cellular proliferation, survival, and resistance to therapeutic agents (22). Everolimus, an mTOR inhibitor, has been assessed both as a monotherapy and in combination with sorafenib. While monotherapy offers limited advantages, combination therapies have shown improved disease control. Preclinical investigations indicate that dual inhibition of PI3K and mTOR may circumvent adaptive resistance, especially in tumors characterized by PTEN loss or PI3K mutations (23). However, the clinical application of these findings is hindered by challenges related to toxicity and efficacy. Current research endeavors are directed toward identifying predictive biomarkers, such as activation profiles and genetic alterations, to enhance patient selection (24). This reflects a shift toward precision oncology in osteosarcoma.

### 2.3 Anti-Angiogenic agents

Angiogenesis is a key driver of osteosarcoma progression and metastasis, rendering it a significant therapeutic target. Apatinib, a VEGFR2 inhibitor, has demonstrated promising activity in advanced osteosarcoma, including partial responses and prolonged disease stabilization, in phase II studies (25). Similar, albeit modest, benefits have been observed with pazopanib and cediranib (26). Resistance to VEGFR inhibition develops rapidly as tumors activate alternative pro-angiogenic pathways or adopt invasive growth strategies (27). To address this issue, angiogenesis inhibitors have been combined with other therapeutic modalities, including checkpoint inhibitors. The normalization of tumor vasculature through VEGFR blockade may enhance immune cell infiltration, thereby potentially increasing the efficacy of immunotherapy (28).

### 2.4 Immune checkpoint inhibitors

**Figure 1:**
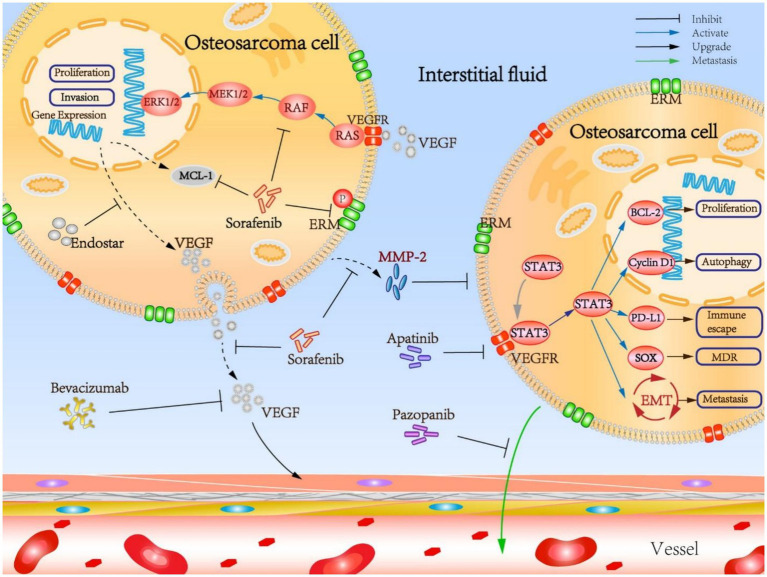
Mechanistic insights into the osteosarcoma targeted anti-angiogenesis therapy. Reproduced from “Mechanistic insights into the OS targeted anti-angiogenesis therapy” by Liu et al. (33), licensed under CC BY 4.0.

Osteosarcoma is renowned for its immunogenic characteristics; however, clinical trials involving immune checkpoint inhibitors have predominantly yielded unsatisfactory results. Pembrolizumab, an anti–PD–1 antibody, was evaluated in patients with relapsed and refractory osteosarcoma, yet exhibited limited efficacy, with most patients experiencing disease progression (29). The obstacles to achieving success include an immunosuppressive TME, a low tumor mutational burden, and the absence of biomarkers to identify potential responders (11). Current research is investigating combination strategies, such as the integration of tyrosine kinase inhibitors (TKIs) with checkpoint inhibitors, chemotherapy, or radiotherapy, to enhance immune activation. Preclinical studies suggest that TKIs, such as regorafenib, may facilitate vascular remodeling to improve T-cell infiltration (30). Furthermore, insights into the TME, including immune suppression and microbial heterogeneity, propose novel strategies for sensitizing tumors to checkpoint blockade (31, 32). Furthermore, anti-angiogenic drugs may contribute to the therapeutic management of osteosarcoma by targeting additional molecular pathways (Figure 1). Table 1 summarizes a comprehensive overview of targeted therapies and immune checkpoint inhibitors in cases of relapsed or advanced osteosarcoma, detailing drug targets, trial phases, patient populations, clinical outcomes, and key references.

**Table 1:** Selected clinical trials of targeted agents and immune checkpoint inhibitors in relapsed or advanced osteosarcoma.

Drug	Targets	Phase	Setting/patient population	Outcome/findings	References
Sorafenib	VEGFR, PDGFR, RAF	II	Relapsed/metastatic osteosarcoma	Stable disease in subset; modest activity	Liu et al. (33)
Sorafenib	VEGFR	II	Relapsed/metastatic osteosarcoma	Improved PFS vs. monotherapy	Flaherty et al.(34)
Regorafenib	VEGFR, FGFR, RET, KIT	II (SARC024)	Advanced/metastatic osteosarcoma	Significant PFS benefit vs. placebo	Davis et al. (18)
Cabozantinib	MET, VEGFR2	II	Refractory osteosarcoma (lung)	Promising activity; manageable safety	Ruiz-Morales and Heng (19)
Apatinib	VEGFR2	II	Advanced osteosarcoma	Partial responses; disease control	Tian et al. (35)
Pembrolizumab	PD-1	II	Relapsed/refractory osteosarcoma	Minimal efficacy; immune evasion	Al Hadidi and Lee (36)

## 3 Emerging strategies and future directions

Targeted therapies offer certain advantages in the management of osteosarcoma; however, sustained responses remain infrequent due to intratumoral heterogeneity, redundant signaling pathways, and mechanisms of immune evasion. To enhance clinical outcomes, emerging strategies focus on combination therapies, biomarker-guided treatment selection, and the development of novel drug delivery systems.

### 3.1 Rational combination therapies

Monotherapies utilizing TKIs, angiogenesis inhibitors, or immune checkpoint inhibitors typically result in transient responses. Rational combinations aim to target multiple pathways. For example, the combination of sorafenib with everolimus has demonstrated superior progression-free survival compared to sorafenib alone, underscoring the value of dual blockade (37). Similarly, the combination of regorafenib with PD-1 inhibitors is under investigation to enhance immune activation by remodeling the tumor vasculature and facilitating T-cell infiltration (30). Preclinical evidence also supports the combination of mTOR inhibitors with chemotherapy, as the suppression of survival pathways enhances chemosensitivity (38). Radiotherapy combined with checkpoint blockade has been proposed to induce immunogenic cell death, thereby potentiating immune responses (39). These examples collectively demonstrate that effective treatment may necessitate the coordinated targeting of both oncogenic signaling and immune evasion pathways (40).

### 3.2 Biomarker-guided and precision oncology approaches

The lack of predictive biomarkers constitutes a notable limitation of this study. Potential candidates, including PTEN loss, PI3K mutations, VEGF expression, and PD-L1 status, have yet to be validated for routine application (41). New methodologies are emerging in this field. Liquid biopsy and ctDNA profiling offer real-time monitoring of clonal evolution and therapeutic resistance (42, 43). Systems-level approaches, such as dynamic network biomarkers applied in thyroid and breast cancers, may capture early molecular shifts predictive of response and could be adapted for osteosarcoma (44). Additionally, heterogeneity in the TME, including immune and microbial diversity, represents a rich source of biomarkers linking stromal biology to therapeutic outcomes (45). These strategies align osteosarcoma research with broader precision-oncology initiatives (46, 47).

### 3.3 Novel drug delivery platforms

The systemic delivery of targeted drugs is impeded by poor bioavailability, toxicity, and limited tumor penetration. Nanotechnology-based carriers have been developed to address these challenges. Liposomal formulations, polymeric nanoparticles, and bone-targeted delivery systems can enhance drug accumulation in tumors while reducing systemic side effects (48, 49). For instance, encapsulating TKIs, such as sorafenib, in nanoparticles improves pharmacokinetics and safety, whereas nanocarrier delivery of checkpoint inhibitors enhances immune cell infiltration and activity. Multifunctional platforms that co-deliver cytotoxic and immunomodulatory agents are also being tested, offering a route to address tumor growth and immune suppression simultaneously (50, 51).

### 3.4 Future clinical directions

**Figure 2:**
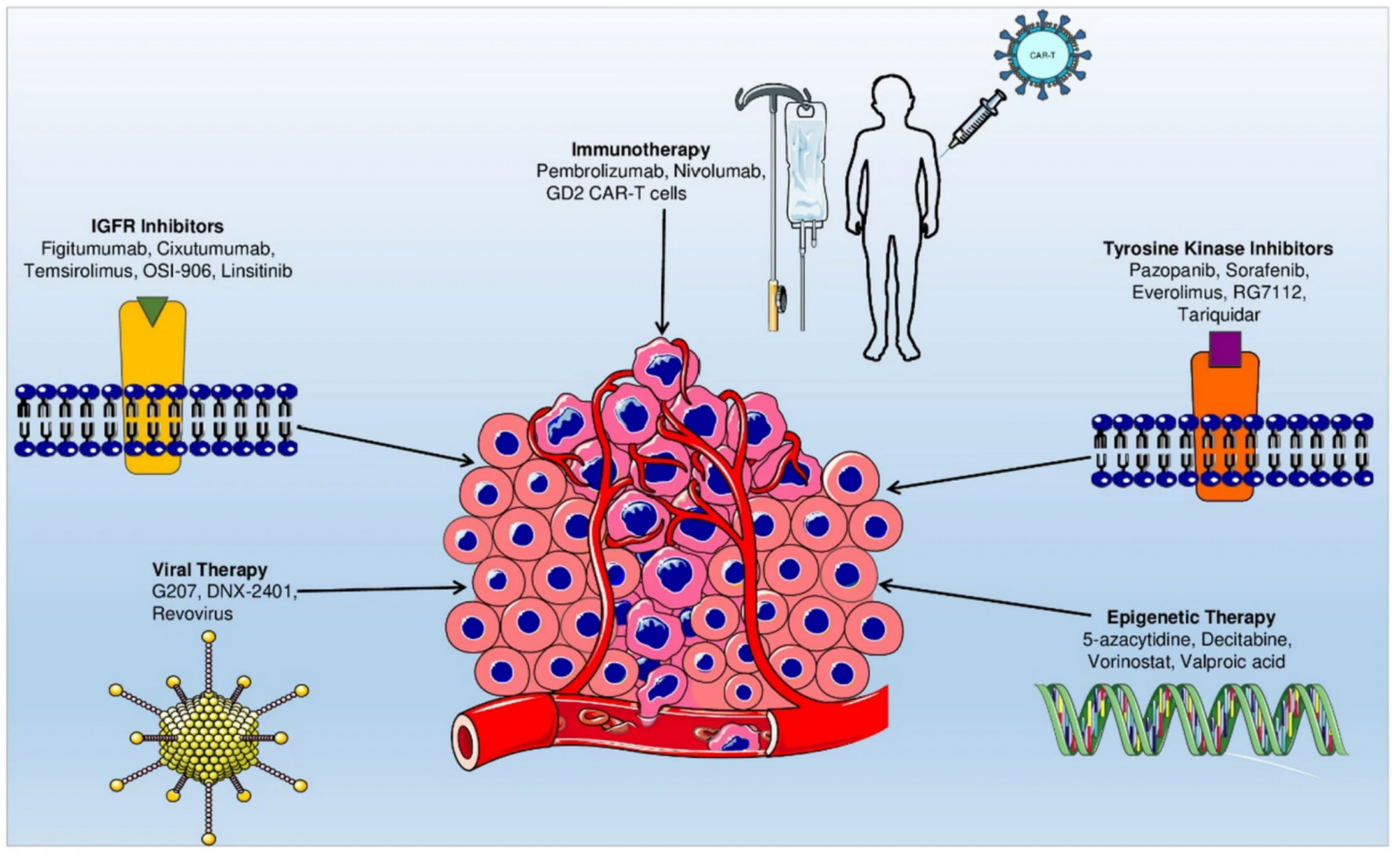
Roadmap of emerging targeted therapeutic strategies for osteosarcoma. Reproduced from “Current advancement in therapies for the treatment of osteosarcoma” by Morya et al. (54), licensed under CC BY-NC-ND 4.0.

The convergence of molecular oncology, immunotherapy, and nanotechnology suggests that osteosarcoma therapy will increasingly rely on multimodal approaches. Adaptive trial designs, such as basket and umbrella trials, have the potential to incorporate real-time molecular profiling to enhance the allocation of therapies (52). Additionally, the integration of biomarkers derived from the TME, including immune signatures, exosomal profiles, and microbial heterogeneity, may improve patient selection (53). To overcome the current survival plateau, it is imperative to foster collaboration among oncologists, molecular biologists, bioengineers, and computational scientists to translate laboratory advancements into sustainable clinical outcomes effectively. Emerging therapeutic approaches, such as gene therapy and oncolytic virotherapy, are currently under investigation. Despite significant advancements, the prognosis for osteosarcoma, particularly in cases involving metastasis, remains poor. This underscores the urgent need for continued research and the development of innovative therapeutic strategies to enhance patient outcomes (54) (Figure 2).

## 4 Challenges and limitations

Despite notable progress in the clinical exploration of targeted therapies, their effectiveness in the treatment of osteosarcoma remains limited by several fundamental challenges. These challenges stem not only from the biological complexities inherent to the disease but also from issues related to trial design, biomarker availability, and safety considerations.

### 4.1 Tumor heterogeneity and genomic complexity

Osteosarcoma is characterized by significant genomic instability, including chromothripsis, structural rearrangements, and extensive copy-number alterations (55). This instability contributes to considerable intratumoral heterogeneity, with subclones possessing distinct oncogenic drivers co-existing within the same tumor. Such diversity results in highly variable therapeutic responses, thereby complicating the development of standardized treatment protocols (56). Unlike tumors driven by recurrent “trunk” mutations (e.g., EGFR in lung cancer), osteosarcoma lacks consistent and targetable mutations. A network of signaling redundancies that enable tumor cells to circumvent the inhibition of a single pathway characterizes the disease. This complexity poses a challenge to the application of a “one-size-fits-all” therapeutic model and underscores the necessity for biomarker-guided precision strategies (57).

### 4.2 Development of drug resistance

Resistance to targeted therapies continues to pose a significant clinical challenge. Both intrinsic resistance, which exists before treatment, and acquired resistance, which develops during therapy, have been documented. These resistance mechanisms encompass compensatory activation of parallel signaling pathways, epigenetic reprogramming, and alterations in the TME (58). For instance, while VEGFR inhibition with agents such as apatinib or sorafenib may initially suppress angiogenesis, tumors can rapidly adapt by upregulating alternative pro-angiogenic factors or adopting invasive growth patterns (59). Drug efflux transporters and metabolic reprogramming contribute to this resistance. Additionally, the immunosuppressive microenvironment, characterized by regulatory T cells, myeloid-derived suppressor cells, and tumor-associated macrophages, can undermine the effectiveness of checkpoint inhibitors (60). The elucidation of these resistance mechanisms highlights the imperative for employing combination regimens and interventions that specifically target the microenvironment.

### 4.3 Lack of predictive biomarkers

A significant limitation in the field of osteosarcoma research is the absence of validated predictive biomarkers. Although various markers, including PTEN loss, PI3K mutations, VEGF overexpression, and PD-L1 status, have been investigated, none have been standardized for clinical decision-making (61). This gap impedes the ability to identify patients who are most likely to benefit from specific therapies, resulting in inconsistent and often unsatisfactory outcomes. Recent investigations into dynamic network biomarkers have demonstrated potential in detecting early molecular changes predictive of therapeutic response. Furthermore, the application of liquid biopsies and circulating tumor DNA (ctDNA) as real-time monitoring tools presents an opportunity to observe clonal evolution and emerging resistance during treatment (44). Nevertheless, these methodologies remain predominantly experimental and necessitate validation in larger, multicenter cohorts.

### 4.4 Limitations of clinical trials

The rarity of osteosarcoma poses considerable challenges in the design and implementation of rigorous clinical trials for its treatment. Most studies are single-arm phase II trials with limited patient enrollment, which restricts their statistical power and generalizability (62). Cross-trial comparisons are further complicated by variations in eligibility criteria, treatment protocols, and endpoints (63). Additionally, traditional trial endpoints, such as progression-free survival, may not adequately reflect the benefits of targeted or immunotherapeutic agents, especially when stable disease, rather than tumor reduction, is the primary outcome (64). Adaptive trial designs and international collaborative consortia are urgently needed to address these limitations and accelerate the translation of promising therapies into clinical practice.

### 4.5 Safety and toxicity concerns

While targeted therapies are generally perceived as more selective than cytotoxic chemotherapy, they are not without associated toxicities. Tyrosine kinase inhibitors (TKIs), such as regorafenib and cabozantinib, are linked to hypertension, hand–foot syndrome, gastrointestinal disturbances, and fatigue, often necessitating dose reduction or treatment discontinuation (65). Similarly, angiogenesis inhibitors can induce vascular complications, including bleeding and thrombosis. Although immune checkpoint inhibitors are typically well tolerated, they may result in immune-related adverse events, such as colitis, pneumonitis, endocrinopathies, and hepatitis. The toxicities associated with treatment are particularly concerning in pediatric and adolescent patients, who constitute the majority of osteosarcoma cases (66). Effective management of these adverse events necessitates multidisciplinary expertise and meticulous monitoring, which may restrict their widespread implementation in resource-limited settings. The challenges highlighted emphasize the complexity of advancing targeted therapies for osteosarcoma. Addressing these challenges requires a deeper understanding of the disease’s biology, the identification of reliable biomarkers, the development of innovative clinical trial designs, and the enhancement of strategies to manage treatment-related toxicities. Collaborative efforts across these domains are essential to improving the efficacy of targeted interventions for this rare and aggressive malignancy (67).

## 5 Conclusion

Osteosarcoma remains one of the most challenging malignancies to manage in pediatric and adolescent populations. Although surgery and chemotherapy constitute the standard treatments, survival rates for relapsed or metastatic cases have not significantly improved in recent decades. The introduction of targeted agents, such as tyrosine kinase inhibitors, mTOR inhibitors, angiogenesis modulators, and immune checkpoint inhibitors, has expanded therapeutic options and demonstrated disease stabilization in certain patients. However, these benefits are constrained by tumor heterogeneity, the rapid emergence of resistance, a lack of predictive biomarkers, and complexities in clinical trial design. Furthermore, treatment-related toxicities present substantial challenges, particularly for younger patients. Current evidence underscores both the potential and the limitations of targeted therapies for osteosarcoma, highlighting the need for innovative strategies that incorporate tumor biology and microenvironmental factors.
